# The Hospital. Nursing Section

**Published:** 1904-12-17

**Authors:** 


### The Hospital.

### Nursing Section.

Contributions for this Section of "The Hospital" should be addressed to the Editor, "The Hospital," Nursing Section, 28 & 29 Southampton Street, Strand, London, W.C.

No. 951.—Vol. XXXVII. *SATURDAY, DECEMBER* 17, 1904.

### Notes on News from the Nursing World.

### OUR CHRISTMAS DISTRIBUTION.

One final word of appeal. Parcels for distribution at Christmas may be sent in up to Saturday afternoon, and we hope that those who may have put off forwarding a contribution until the last moment will remember that it is not too late to purchase some little addition to the collection. The need is very urgent; it was never more so. Here is another letter just received, which shows how anxiously some of our readers are awaiting the receipt of a parcel. The writer is the matron of one of the principal London hospitals, and she says "It is with grateful remembrance of the nice bundle of warm garments you so kindly sent to me last Christmas for the patients of this hospital, that I again venture to approach you with the hope that we may participate in your distribution of this year. Thanking you in anticipation." It, of course, depends upon the generosity of our friends how far we shall be able to respond to such applications as this. The whole of the articles received will be on view at the offices of The Hospital on Tuesday afternoon next, and nurses, or other contributors who desire to see them will be welcome any time between 3 and 5 p.m. We have to acknowledge the receipt of parcels from F. D., Westgate-on-Sea, and Miss E. Smith, 15 Percy Road, North Finchley. All contributions should be addressed to the Editor, 28 and 29 Southampton Street, Strand, London, W.C., and should have "Clothing Distribution" written outside them.

### MISS FLORENCE NIGHTINGALE AND SOUTH LONDON NURSES.

There was a large gathering last week of friends and supporters of the South London District Nursing Association, on the occasion of the annual At Home, when collecting cards were received by Mr. Leonard Darwin. Mr. John Burns, M.P., said that on behalf of his large working-class constituency, he could testify to the educative effects of the nurses' visits, 36,932 of which were paid last year. The following letter from Miss Florence Nightingale was read:—

Dear Miss Bullock.—Miss Florence Nightingale has much pleasure in sending a cheque for £5 for the South London District Nursing Collecting Card Fund. It is a subject she has very much at heart, and she feels strongly that the work of a district nurse neither begins nor ends with nursing only. By her influence and teaching on sanitary and other matters a good and sympathetic woman will educate the people among whom she works. They must be taught to do as much as possible for themselves, and many intelligent and willing probationers will be found among the patients' friends—who will learn to make the best use of the means within their reach. Miss Nightingale desires to send her best wishes for the useful work of your excellent Association.—Believe me, yours truly,

Elizabeth Bosanquet, Secretary.

### PRINCESS LOUISE AND THE QUEEN'S NURSES AT EDINBURGH.

During her stay in Edinburgh last week Princess Louise, Duchess of Fife, paid a brief visit to the Scottish District Training Home of Queen Victoria's Jubilee Institute in Castle Terrace. Her Royal Highness presented the Queen Victoria Jubilee badges and certificates to the 16 nurses present who had qualified during the year. Subsequently, the Duke of Argyll, replying to a vote of thanks proposed to the Princess, congratulated the nurses upon having taken up the work, and said that in the calling they had to pursue they would always be grateful for the time they had spent and the training they had received as Jubilee nurses. A number of young girls were afterwards presented to the Princess as representing the schools in the city which subscribe for the upkeep of the Cowgate nurse.

### PENSIONS FOR GLASGOW NURSES.

Following the example of the managers of the Victoria Infirmary at Glasgow, the members of the Glasgow and West of Scotland Co-operation of Trained Nurses assented at their annual meeting to a resolution in favour of establishing a pension fund for their nurses. The proposal in each case is to adopt a scheme of federation to the Royal National Pension Fund, half the contribution being paid by the institution and half by the nurses themselves. Mr. Cameron Corbett, M.P., in strongly advocating the proposal, expressed his earnest hope that all the infirmaries in Glasgow would move in this matter, and Mr. J. D. Hedderwick expressed a similar opinion. We are justified in regarding this as a first step in the largest scheme of federation with the Fund yet attempted, and we earnestly hope, for the sake both of Glasgow nurses and also for that of the institutions with which they are connected, that the movement will be speedily carried to a successful issue.

### POST-GRADUATE CERTIFICATES AT BRISBANE HOSPITAL.

Several improvements have been made in the regulations for nurses at Brisbane Hospital. The minimum age of probationers has been advanced from nineteen to twenty years. The new minimum is, of course, too low, according to the English standard, but it is satisfactory that there has been a step forward. If a nurse who has completed her three years' training shows special aptitude she will be recommended for a further engagement of twelve months, during which she will rank as a staff nurse, receiving a salary of £36. In this year she will receive additional instruction, both theoretical and practical, and at the end of the year will be further examined. If she be then successful she will receive a post-graduate certificate. It will be interesting to observe whether the introduction of a post-graduate certificate is appreciated and whether it increases the popularity of the training school. To be of much value, however, it should show that the possessor has had experience in private and special nursing.

### THE REHABILITATED CERTIFICATE.

We were able last week to report that the Croydon Guardians had at length restored to the certificate, given to the probationers of their training school, its value. The withdrawal of the matron's signature four years ago was first condemned in our columns and we are glad to know that the attitude of The Hospital, which was for a long time denounced by the Guardians, had a great deal to do with this ultimate renunciation of a mistaken policy. Dr. Addison, who naturally recognised the folly of reducing the value of the certificate of the Croydon Infirmary, and the injustice of not giving probationers trained in the institution the same advantages afforded elsewhere, has been unremitting in his efforts to get the matron's signature back to the certificate. He is particularly entitled to the gratitude of the Guardians for proving himself so fully alive to their interests as well as to those of the nurses.

### A QUESTION OF CONFIDENCE.

The religious question has cropped up at Maryport, this time in the case of the appointment of a district nurse. It is complained that one of the candidates was asked whether she was a Churchwoman and that because she was obliged to say she was a Nonconformist, she was not elected. The rejoinder of the committee is that another person was chosen because she had enjoyed greater experience, and not because she was a Churchwoman. If the subscribers have confidence in the committee, they will accept this statement as accurate; if not, it is open to them to change the committee.

### LORD CURZON'S GRATITUDE.

It will be remembered that during her illness at Walmer Lady Curzon was nursed by the matron of the Victoria Hospital at Deal and members of the private nursing staff. Before his departure to India Lord Curzon sent a cheque for £50 to the Victoria Hospital. In his covering letter he said that he should like to make a slight acknowledgment of the great kindness that had been shown to him in the neighbourhood, and notably by those who had anything to do with "this terrible illness," by offering a small donation to the funds of the excellent local hospital, by the help of whose admirable superintendent and nurses, and resources in general, he had profited so much.

### CHRISTMAS PREPARATIONS IN THE LONDON HOSPITALS.

Little, if any, deviation from the customary Christmas festivities at the London hospitals will take place this year. Christmas Day being on Sunday, most of the festivities will be a day earlier or later, and at the Middlesex Hospital, for example, we learn that Christmas Eve will be the great day, while, as usual, the children's parties in Princess May and Percy Wards will be held during Christmas week. At the London, Guy's, St. Thomas's, and King's College Hospitals it is quite a well-established custom for the students to form themselves into voluntary bands of entertainers, and the preparation of plays, character-sketches, etc., is well in hand long before Christmas or Boxing Day, when the wards are visited in turn by the various troupes. At Guy's, too, the out-patient treat is a perennial institution, and is greatly looked forward to by hundreds of children in the Borough. A time-honoured custom at the Royal Free Hospital is the singing of carols by the lady students on Christmas Eve, when Chinese lanterns are carried through the wards with very pretty effect. At St. Bartholomew's Hospital the work of entertaining is in the hands of each ward-sister, and occupies the greater part of Christmas Day, tea-time being the great time for all concerned.

### PAYMENT OF CHRISTMAS INSTALMENTS OF PENSIONS.

Hitherto, the Royal National Pension Fund for Nurses, following the custom of Government departments and commercial undertakings, has made remittances for the Christmas quarter the day before Christmas Day. There is no doubt that at this season of the year even people with very small incomes desire to add to their purchases. But the receipt of a cheque on Christmas Eve is particularly awkward, as the money cannot be obtained for two days owing to Bank Holiday succeeding Christmas Day. We are therefore glad to be able to announce that, bearing this in mind, and in accordance with their invariable practice of taking the interests of all their policy-holders into consideration, the Council have decided that in future the Christmas instalments shall be sent out on December 20th, and thus be in the hands of the annuitants three clear days before Christmas. Any of the latter who have changed their addresses should advise Mr. Louis H. M. Dick, Secretary of the Pension Fund, 28 Finsbury Pavement, E.C., before Tuesday next.

### HOSPITAL FOR INVALID GENTLEWOMEN.

The two days' sale of useful and fancy articles in aid of the funds of the Hospital for Invalid Gentlewomen was opened at 90 Harley Street, W., on Tuesday. In the absence of Princess Victor of Hohenlohe, the ceremony was performed by Lady Maud Wilbraham. Dr. John Phillips drew attention to the fact that this was the first time the committee had resorted to the expedient of a sale of work to meet the current expenses of this small but useful institution, which he referred to as an institution doing a quiet work out of the public gaze. Lady Maud Wilbraham having declared the sale open, Dr. G. Lenthal Cheatle proposed a vote of thanks, coupling with Lady Wilbraham's name that of Mrs. Laxton Noble, to whom the hospital was indebted for the gift of a steriliser. The work, some of which was done by the nurses, was attractively displayed on eight stalls; the various stallholders, among whom was the Honourable Victoria Grosvenor, being assisted by the nurses attached to the hospital.

### THE VALUE OF AN AUTOGRAPH QUILT.

Many ingenious methods are adopted to raise funds for District Nursing Associations. To the ladies' committee at Dartmouth belongs the credit of having carried through a scheme for an autograph quilt, which they made themselves from the squares on which the autographs had been placed. The signatures obtained included those of Princess Beatrice and her daughter, the Premier, the Lord Chancellor, Mr. Joseph Chamberlain, the Chancellor of the Exchequer, the Bishops of London, Liverpool, Richmond, and Stepney, and a host of other notable persons, each of whom contributed not less than 1s. for the privilege of signing their names. In addition to the autographs, the Borough Arms were worked in the centre of the quilt. Another lady worked the lace which fringed the quilt, which measured 12¼ yards round, and this task occupied her for 256 hours. The labour of love by all met with a substantial reward, for at the auction 11 guineas were given for the quilt. As the receipts for the autographs were about £30, over £41 was thus added to the funds, irrespective of the amount realised by the concerts which took place on the day of the sale.

### THE VALUE OF FARTHINGS.

A successful At Home, at which a large number of guests were present, was given last week by the lady superintendent, Miss Curtis, and the nurses of the Hammersmith and Fulham District Nursing Association. The Mayor of Hammersmith presided, and the principal speaker was Mr. W. J. Bull, M.P., who pointed out that while the population of the district amounted to nearly 238,000, the Association was hampered for want of an additional £200 a year, and if everyone living in Fulham and Hammersmith would give one farthing a year, the nurses would be out of debt and difficulty. In contrast to this lack of sufficient local support, he mentioned that the sum of 7s. 6d. in farthings had been collected by the infants in one of the local schools, the mistress of which wrote that the nurse was one of their most welcome visitors. It was amusing, she added, to see how the children looked themselves over on the days when the nurse was coming, to see whether there was not something the matter with them, so that they might be treated by her. Another farthing collection amounted to 9s. 6d., and the total amount received in the collecting boxes and cards, was £99 3s. It was stated by the Chairman that a scheme was on foot by means of which it was hoped the finances of the Association might be greatly augmented.

### PRIZE DAY AT BRISTOL CHILDREN'S HOSPITAL.

The presentation of prizes and certificates of merit to the nurses of the Royal Hospital for Sick Children and Women at Bristol is always an interesting function. At the ceremony this month, the president of the hospital was in the chair, and the matron, Miss Mattick, read the list of the awards. Among second-year nurses, the prizes for anatomy and physiology and for medical nursing were won by Miss White; and among first-year nurses, Miss Eleanor Richards was equally successful. Of second-year nurses, Miss Thompson and Miss Corbett were awarded certificates of honourable mention for anatomy and physiology, and Miss Elkins and Miss Corbett for medical nursing; of first-year nurses Miss Newton, Miss Richards, and Miss Gotto obtained certificates for anatomy and physiology, and Miss Brough was successful in medical nursing. The matron, in proposing a vote of thanks to the wife of the chairman who presented the prizes and certificates, asked the nurses, whilst endeavouring to make the hospital second to none in the training of nurses for sick children, according to the most approved and up-to-date methods of surgery and medicine, not to forget to look at their work from a womanly and sympathetic, as well as a scientific standpoint. She begged them, although all loved what were called interesting cases, not to think anything too trivial or too tiresome to do for any patient, however chronic the case might be, if it would only alleviate suffering in the slightest degree.

### SOCIAL MEETING AT NOTTINGHAM.

An informal but pleasant little gathering of some of the nurses and others interested in the Notts Nursing Federation, including its honorary secretary, Miss Vickery, was held on Saturday last by the invitation of Miss Ross, county superintendent, in the parish room of St. Andrew's Church, Nottingham, kindly lent by the vicar for the occasion. The weather was cold and cheerless in the extreme, and unfortunately prevented some from being present, but the room looked delightfully bright with its cheerful fires, dainty little tea-tables, and pretty flowers. Excellent vocal and instrumental music was provided by friends, and was much appreciated by all. Miss Amy Hughes, superintendent of County Associations, said a few words to the nurses, both Queen's and Village, who were present, pointing out the privileges, and yet at the same time the grave responsibilities, of their special work. The impromptu afternoon was a great success, and the guests left reluctantly when "train times" made speedy departures necessary if districts were to be visited that evening.

### CONCERT AT GUY'S HOSPITAL.

An excellent concert by the Nurses' Choral Society and the Students' Glee Club took place in the Court Room of Guy's Hospital on Tuesday evening last. The nurses were all in uniform and arranged in two groups—the contraltos on the right, and the sopranos on the left—on the platform which was prettily decorated with palms. The students ranged themselves behind the nurses. There was a good audience, and a varied and attractive programme was admirably gone through.

### SHORT ITEMS.

Mrs. L. Mooijacul (*née* M. B. Horswell) has been appointed Honorary Serving Sister of the Order of St. John of Jerusalem in England. She was trained at Salisbury Infirmary, and was afterwards sister at Taunton Hospital, matron of the Burgher Camp Hospital, Klerksdorp, Transvaal, and matron of the Belfast Civil Hospital, Transvaal.—The East End Mothers' Home has been approved by the Central Midwives Board as an institution for the training of midwives.—Miss L. Belcher, Miss K. A. Allsop, and Miss A. Rowe have been provisionally appointed staff nurses in Queen Alexandra's Imperial Military Nursing Service.—About 25 nurses from the neighbourhood of Taunton met at the Grange, Kingston, on Thursday last week, to hear an address from Dr. Peuron Williams on "The Influence of the Mind on the Body."—The Society of Trained Masseuses will hold examinations at 12 Buckingham Street, Strand, on Saturday, January 28th; Friday and Saturday, February 3rd and 4th; and Monday and Tuesday, February 6th and 7th. Forms of application must be filled in and returned not later than January 9th.

### The Nursing Outlook.

"From magnanimity, all fear above;
From nobler recompense, above applause,
Which owes to man's short outlook all its charm."

### A NURSE'S SAVINGS.

The phenomenal success of the Royal National Pension Fund for Nurses has had a marked effect upon the policy of a good many insurance offices in regard to annuities. Formerly deferred annuity business was not done to any great extent, but in recent years several insurance offices have given special attention to this branch of business. So great has been the success of the Pension Fund that its invested funds will soon amount to one million pounds sterling. Hence the attention which this exercise of thrift on the part of nurses has excited, and the occasional attempts made, as a matter of business, by a very few offices, we are glad to say, to induce nurses to place their savings with them on less advantageous terms than those which the Pension Fund can offer from its mutual character and organisation.

The latest attempt of the kind has been made by a Scotch assurance company, which heads its prospectus "Benefits for Trained Nurses." We have thought it right, for the information of nurses, to obtain a report on this scheme from the consulting actuary to the Pension Fund, which will be found in another column. In that report Mr. King shows by actual figures, and for reasons given, that nurses will be wise in their own interests to support their own Pension Fund, instead of going to an insurance office like the one in question. Amongst the advantages of the Pension Fund is that for a slightly less premium it will grant a nurse a larger guaranteed pension at the age of 50 than the insurance office, which not only does not guarantee the pension at the amount stated, but is likely to reduce the amount rather than increase it, because companies are stiffening their annuity rates at the present time.

The actual example taken is that of a nurse aged 25, who would pay the insurance company by quarterly sums £12 11s. 4d., as against £12 11s., the monthly payments rate of the Pension Fund. For these payments a nurse would enter for a non-guaranteed pension in the insurance office of £24 18s., whereas in the Pension Fund she would get a guaranteed pension of £26 5s. 5d., the pensions commencing in both cases at 50 years of age. For about the first 13 years the company would make larger payments on death than the Pension Fund, but after that period the Pension Fund's payments on death would be much larger than the insurance company, as shown in Mr. King's report. Nurses, however, do not desire to invest their savings to provide for their friends after their death, but to secure a provision for themselves against the day of sickness—for which the insurance company offers no facilities—and on retirement from work. It will be no inducement, therefore, to an intelligent nurse, we imagine, to be told that, if she dies within thirteen years or so of her commencing to save, the insurance office may give her executors a larger sum than the Pension Fund, especially when she realises that such a step would cut her off entirely from the special protection and advantages of her own fund, which she is always guaranteed, from the commencement of her first payment to the Royal National Pension Fund for Nurses.

Thus the Pension Fund offers the nurse sick pay; it entitles her to a share in all the profits of every kind, which are exceptionally large, because the expenses of management are so small; it entitles her to borrow on advantageous terms; and to enjoy the substantial help of the benevolent branch, should she ever need it, throughout the whole period of her professional career. To state these special gains is merely to show, what every nurse can readily realise, that her own fund offers much greater advantages than can be secured at any insurance office. Indeed, every nurse who joins the Pension Fund for sick pay and pension, may consider herself safe for the rest of her life. Nurses are beginning, too, to understand the advantages of co-operation, and no nurse who respects her profession and herself will fail to understand that the larger the number of nurses who enter the Pension Fund, the better it must be for every policy-holder who entrusts her savings to it.

In our view, as we have always maintained, it is wise for every nurse to enter for sick pay, as well as for pension. Sick pay has been the comfort of a large and increasing number of Pension Fund nurses in recent years. The Pension Fund requires no medical examination for pension, but only for sick pay, and we are surprised to learn, that nurses, of all people, are inclined to dread a medical examination, which, in the case of a healthy nurse, should be a matter of mere routine.

Finally we are glad to know that an increasing number of hospitals have associated themselves with the Pension Fund and are paying a portion of the premiums entitling the nurses on their staff to pension allowances. We hope, that within the next 10 years, there will not be a single great hospital in the empire which has not adopted this plan, for it is just to the nurse, economical to the institution, and honourable to both. Through its connection with the Pension Fund, Guy's Hospital, for instance, has secured to each of its nurses, who are engaged in private nursing, a retiring allowance, which, on the average, will probably exceed £60 a year. That is a pleasing fact to record, when certain hospitals still farm their nurses and make a large profit out of their work, without sharing these profits with their nurses as they in fairness ought to do.

### Lectures upon the Nursing of Infectious Diseases.

By F. J. Woollacott, M.A., M.D., B.S.Oxon, D.P.H., Senior Assistant Medical Officer, Park Hospital, Metropolitan Asylums Board, Hither Green.

### LECTURE XI.—DIPHTHERITIC CROUP.

One of the most dangerous complications of diphtheria is extension of the disease to the larynx and trachea, for the presence of membrane in these situations obstructs the entry of air with consequent risk of suffocation. The symptoms are readily understood if we bear in mind the anatomy of the parts. The larynx, situated just above the trachea, contains the vocal chords which are set vibrating by the air current and so produce the voice. They consist of two fibrous bands, one on each side, which by the action of various muscles may be separated or brought close together, but even when widest apart they always to some extent narrow the passage. It is easy to see therefore that, when they are attacked by disease, two results must follow. The voice will be altered in character and the respiration will be embarrassed. In diphtheria the chords are first inflamed and then covered with membrane, which may then invade the trachea and extend to the lungs. As a rule the membrane spreads down from the throat, but sometimes it makes its first appearance in the larynx.

The earliest symptom is cough, which rapidly becomes hard, hoarse and croupy. The inspiration is difficult and attended with a peculiar noise, known as stridor, while the voice loses its clearness and grows weak and husky, until it may be reduced to a mere whisper. At the same time other symptoms appear. Owing to the violent efforts made to draw in air through the swollen and obstructed larynx the soft parts about the chest are sucked in at each inspiration. The younger the patient the more marked is this, and when the bones are soft and yielding there is deep recession of the lower ribs as well as of the abdominal wall and neck. The face is pale and, as the obstruction increases, the lips become blue owing to the deficient aeration of the blood. The patient is then restless, fighting for breath, throwing himself about in bed, and from time to time clutching at his throat as if trying to tear away the obstruction. His expression is anxious and terror-stricken and the face bathed in perspiration. Unless relief be afforded he is soon worn out with his struggles. The pulse grows weak, the cyanosis deepens, the inspiratory efforts become more and more feeble, until he sinks into a comatose condition ending in death.

From this description it will be seen that there are three well-marked stages, the first characterised by croupy cough, but only slight discomfort, the second by gradually increasing restlessness and struggling for breath, and the third by exhaustion and coma. Whatever be the patient's condition and however mild the symptoms, he must always be carefully watched, for at any time he may become suddenly worse, so that prompt treatment is necessary to save his life. Not infrequently these sudden acute attacks pass off after a time and are succeeded by a period of comparative rest or even sleep, but they are very apt to recur and may prove fatal. We must therefore not be deceived by a transient improvement.

In the treatment of diphtheritic croup the administration of antitoxin is invaluable, and without it the other means at our disposal will often be of little use. It should be given in a large dose and as soon as possible. As regards the rest of the treatment we are guided by the urgency of the symptoms. If the disease be in the earliest stage or not far advanced in the second we should proceed as follows. A hot bath should be given and will often afford considerable relief. Afterwards the patient should be put to bed, kept quiet and encouraged to sleep, so that the antitoxin may have time to act. The bowels must be opened by an aperient or better still by enema. The temperature of the room should be 65° F., or a little over, and the bed must be protected from draughts. If the air be very dry, benefit may result from the use of the steam-kettle. The old-fashioned steam-tent is occasionally useful, but is less often ordered than formerly. Its chief disadvantage is that it shuts in the bed too closely so that the air, though moist, is apt to stagnate, and become close and foul. Moreover it keeps out the light and interferes with the nursing and examination of the patient. A better plan is to arrange a screen round the head of the bed, roofed in at the top with a sheet, forming a half tent into which the steam can be directed. By this device the air is moistened quite sufficiently, and at the same time always remains fresh, while as much light can be admitted as is required. Our object in using the steam is to relieve the laryngeal spasm, so frequently present, to soothe the inflammation, and to assist in the separation of the membrane. The food should be liquid and chiefly milk, given in small quantities and at frequent intervals; but if the patient is sleeping he should not be awakened. At this stage rest is more important even than nourishment. It often happens that with this treatment the symptoms gradually subside and recovery follows. On the other hand they may get more urgent and render an operation necessary. This is always required if there be increasing restlessness and difficulty of breathing, or if signs of exhaustion have appeared. There are two operations that may be performed. In the first a tube is passed through the mouth into the larynx so as to make a way through the obstruction; in the other the windpipe is opened and a tube inserted below the obstruction. The former is known as intubation, the latter as tracheotomy.

(*To be continued.*)

### Sick-Room Help Society.

Through the kindness of Sir Edward and Lady Sassoon a very successful drawing-room meeting was held at their house, 25 Park Lane, last week, in aid of the Sick-Room Help Society.

The meeting was addressed by Sir Edward Sassoon, the Rev. S. Singer, Miss Löwry, Mrs. Model (president and hon. secretary), Mrs. Nathaniel Cohen, Mr. Leonard Cohen, the Rev. A. A. Green, and Mr. Harry S. Lewis, of Toynbee Hall.

Mrs. Model spoke of the sanctity of motherhood, and said that in the potentialities of the infant lies the strength of the ages to come. And this, not only in well-born children, but also in those other babies born daily in the slums and crowded ghettos. She begged that some of these might have their early chance. Alluding to the registration of midwives, she expressed the opinion that there was no question of the society trenching on their ground. "We have now," she continued, "five trained nurses and 36 'helps,' but several more trained women are needed. Lately, as many as 60 or 70, or even 80, mothers and babies have been attended weekly, each case being nursed for a fortnight."

The Rev. A. A. Green especially appealed for funds, which are urgently needed, and after his speech there was a short interval for a collection, which resulted in over £100 being subscribed.

Mr. Harry S. Lewis spoke of the indirect as well as the direct good done by the society. He also referred to the terribly high rate of infant mortality amongst the poor.

### "Benefits for Trained Nurses."

UNDER this heading a Scotch assurance company has issued a prospectus embodying a scheme to secure a fixed sum payable to a nurse in cash at a given age, which, at her option, can be devoted to the purchase of an annuity and a guaranteed sum to her executors in the event of her death before that age is attained. In years past we have frequently had to examine schemes of this kind, and it has been invariably shown that they in fact do not offer nurses anything like the same benefits which they can obtain for the same payments in the Royal National Pension Fund for Nurses. The reason for this is, as all the leading actuaries in this country admit, that the mutual character of the Royal National Pension Fund, its large and special funds, and the system under which it is worked, secure to the nurses the whole of the profits of the business, and many other advantages, which it is impossible for any insurance office, as a mere matter of business, to offer for the same rates of payment. Mr. George King, the consulting actuary of the Royal National Pension Fund, whose devotion to the interests of nurses has been shown over and over again during many years past, has been kind enough to make the following report on the scheme offered to nurses by the Scotch Insurance office referred to. His report is dated the 5th inst., from 15 Walbrook, London, E.C., is addressed to the secretary of the Pension Fund, and is as follows:—

### ASSURANCES FOR NURSES.

I am in receipt of your letter of to-day's date, and now return the prospectus which you sent me.

The assurances granted by the company in question are simply double endowment assurances without profits, and the benefits they afford to nurses cannot for a moment be compared with those offered by your Fund. It will be well to take up the actual example given in the prospectus, viz., that of a nurse age 25 taking out an assurance for £400 payable at age 50 with an assurance of £200 payable should she die before that age, the assurance for £400 at age 50 being convertible at option into a pension which is said to be £24 18s. The annual premium for this benefit is £11 19s. 10d., but if we take the quarterly premium which is more comparable with the rates of the Pension Fund the premium per annum is £12 11s. 4d. It should be noted that the pension of £24 18s. is not guaranteed and that in a footnote it is stated that the pension will be calculated at whatever may be the then rates of the company. The pension of £24 18s. is, therefore, likely to be reduced and is not at all likely to be increased, because companies at the present time are stiffening their annuity rates.

For a premium of £1 0s. 11d. per month or £12 11s. per annum, the Pension Fund will grant to a nurse at present aged 25 a guaranteed pension of £26 5s. 5d. per annum to commence at age 50, and that pension would share in surplus, and would certainly be considerably increased. Should the nurse, during the 25 years before the pension matures, desire to surrender it, she will get back her money with interest; and should she die before the pension matures, the money would also be returned to her representatives with interest. Comparing these returns, for about the first 13 years, the Company in question would make larger payments on death than the Pension Fund, but, after about 13 years, the Pension Fund would make larger payments on death than the Company.

The following are the figures:—

*Payments on Death before Pension.*

| Years in Force. | Pension Fund. | Company. |
|---|---|---|
| | £ s. d. | £ |
| 5 | 63 13 1 | 200 |
| 10 | 142 7 1 | 200 |
| 15 | 227 17 2 | 200 |
| 20 | 324 11 7 | 200 |

On maturity the payment by the company would be £400 in lieu of pension, and the Pension Fund would pay £434 0s. 7d. Thus, while for the first few years the company would pay rather more on death than the Pension Fund, yet afterwards it is all the other way about, and the Pension Fund has a great advantage.

When, however, we come to the question of surrender, the advantages of the Pension Fund are incomparably greater than those of the company. Nothing is said in the prospectus about surrender value, but in the article in the *British Journal of Nursing* it is said that the nurses under the company's scheme would have the "assistance" of being compelled to exercise thrift. That can only mean that in the event of her wishing to cease her payments she would make a loss. With the Pension Fund, however, there is not this compulsion, and the nurse would not make a loss, but, as mentioned above, she would receive her money back with interest. I cannot say exactly what the surrender value of the policies of the company would be, but I can give an approximation which is probably in excess of the truth. Taking such approximate values, the following is a comparison of the figures:—

*Payments on Surrender before Pension.*

| Years in Force. | Pension Fund. | Company. |
|---|---|---|
| | £ | £ |
| 5 | 63 13 1 | 20 14 6 |
| 10 | 142 7 1 | 51 14 3 |
| 15 | 227 17 2 | 90 1 4 |
| 20 | 324 11 7 | 139 6 10 |

It would be incomprehensible to me that a nurse with the above facts before her should prefer the Company to the Royal National Pension Fund.

Yours faithfully,

GEORGE KING.

∴ An article on "A Nurse's Savings" will be found on page 167 of this number of the Nursing Section of the HOSPITAL.—ED. THE HOSPITAL.

### Opening of a Nurses' Settlement at Clapton.

ON Saturday afternoon Princess Christian, President of the Royal British Nurses Association, opened No. 20 Clapton Square, Hackney, as a settlement for members disabled in the pursuit of their calling or from other causes wishing to retire from active work.

The house, a four-storeyed building, stands at one corner of the square, and consists of a series of bed-sitting-rooms, three on each floor, with the exception of the basement and entrance, the former of which contains the sitting-room devoted to the inmate in charge—who has a separate bedroom on the top floor—scullery, cupboards, etc., while on the entrance floor one room has been set apart as an office. The accommodation consists of a bed-sitting-room for each inmate, and there are three lavatories but no bath-room, as it has not so far been found possible to include this very desirable feature. Each room is furnished with a small kitchen range and a cupboard, one-half having shelves, while the remainder forms a hanging press. Residents will provide their own furniture. The colour chosen for the walls is a blue-grey, and linoleum of the same tint has been laid on the floors. In order to constitute the inmates tenants, a nominal rent of one penny a week will be charged, and the same moderate sum will provide for medical attendance from medical officers appointed by the committee, or dispensary medical officers. In cases of protracted or serious acute illness, however, residents will be removed to the hospital or infirmary upon the doctor's certificate. It is expected that the cost of gas may be covered by a charge of 6d. per week per head, and coal, which will be weighed out weekly and carried up to the several rooms, will be charged at cost price. A moderate income—between seven and fifteen shillings per week—is required. The inmates will be expected to consider themselves bound to study the general comfort and happiness of the other residents, and in the event of sickness to be willing to help as much as possible. Notice of intended absence of more than four days must be given to the lady visitors, and an absence of more than six weeks in the year without permission from the Board of Management will render a resident liable to forfeit her room.

### Everybody's Opinion.

### WOMEN ON BOARDS OF MANAGEMENT OF HOSPITALS.

Lady Hermione Blackwood writes from Clandeboye, County Down, Ireland: I am convinced that I am expressing the feelings of many besides myself when I say that the article in The Hospital on the subject of women as members of hospital boards filled us with regret and dismay. It must seem to us an anomalous thing that a paper such as yours, primarily intended for women, and supported chiefly by women's subscriptions, should use its pages to advocate their exclusion from positions of the kind. Is it not an undisputed fact that the enormous improvement begun in workhouses and workhouse infirmaries forty years ago was entirely due to the initiative of a woman, and is it not mainly owing to the women who are treading in her footsteps now that that improvement is continuing? Why should it be otherwise with hospitals? If it is true that there sometimes appears to be more friction on boards where women have a seat, has it never occurred to you that this may be accounted for by the very fact that a woman detects failings in management which escape men's notice, and also that it is far easier for a matron to impose her views upon a man's committee than upon one in which women have a voice? Your suggestion that women should be allowed and encouraged to collect for hospitals appears to us positively amusing when, in the same breath, you assure us we are not worthy to have a say in the spending of it.

[We gather from her letter that Lady Hermione Blackwood cannot possess intimate knowledge of the inner working of one of the best-managed great modern hospitals. Nowadays it is not true of such hospitals as a whole—though there may be an exception, the details of which we should like to have if it really exists—that it is possible for a matron to impose her view's upon a man's committee, for the sufficient reason that where all are earnestly working to secure the general efficiency, evils of this kind disappear. In the past there may have been something in such an objection, but to-day a modern hospital requires the services of women of character and education, in its higher offices especially, who possess technical knowledge, and for this reason no amateur lady could usefully or effectively sit upon a hospital board, especially if her governing principle is that a woman "must detect failings in management which escape men's notice," and that it is her duty "to prevent the matron from imposing her views upon the committee." In these days matrons and superintendents of great hospitals are selected for their knowledge and capacity, and when appointed they are supported in those positions unless they prove themselves to be incompetent or unworthy. Moreover, we are convinced that no true woman will withhold her subscription from a well-administered voluntary hospital because she is not elected to a seat on the committee of management.—Ed. The Hospital.]

### Christmas Novelties.

By Our Shopping Correspondent.

(*Continued from page* 161.)

### EMBROIDERY AND LACE.

The art of mercerising cotton has placed it in competition with silk, not only in dress materials, but in fancy work also. Some samples of embroidery yarn which I have before me emphasises this fact. These coloured embroidery cottons are beautiful in tint and smooth and lustrous as silk. They are, moreover, very strong, wash well, and are much cheaper than their rival textures. Messrs. Copley and Marshall, of New Mill, Huddersfield, the manufacturers, have also a beautiful lace thread of that light string or biscuit colour which is so popular. The yarns and threads are made in a very large number of shades and various thicknesses.

### BUTTER SCOTCH.

Everyone knows Callard and Bowser's Butter Scotch, and it seems almost unnecessary to mention so old a friend; but at this time of the year there are so many claimants for notice that any good thing may be overlooked. I have one of those neat and inexpensive little packets of the excellent sweetmeat before me, and it occurs to one how suitable such a gift would be on the Christmas tree or in the bran pie, both of which must be catered for at Christmas. Both young and old are likely to be pleased if a packet of Butter Scotch falls to their lot.

### MESSRS. MAPLE'S CHRISTMAS CATALOGUE.

Very many of our readers must shop exclusively by post, and to such we would recommend the attractive and comprehensive catalogue of Messrs. Maple of Tottenham Court Road. Therein they will find suggestions for presents suitable to the means of rich or poor, and such as will please all kinds and conditions of men. Amongst the novelties of the season are all kinds of articles for the writing-table in a tapestry covering, which is most effective and artistic. The great variety of postcard albums calls for notice, whilst every imaginable fancy trifle is to be found side by side with the most useful and durable articles, such as travelling-trunks, clocks, decorative furniture, and silver goods.

### CHRISTMAS BAZAAR AT MESSRS. GARROULD.

Messrs. Garrould's establishment in Edgware Road is visited by so many nurses resident in London that the attractions of their Christmas bazaar have probably been discovered before now. But to those dwelling in the country the illustrated list of some of the numerous articles which appeal to Christmas givers will prove most suggestive. The selection of mechanical toys is especially large and tempting. There are numerous useful articles amongst the fancy leather goods, and the writing and toilet tables could be charmingly furnished at small expenditure. In fact, there is something for everybody to receive and for everybody to give.

### Where to Go.

Pictures of Norway.—The collection of charming water-colour drawings of scenes in Norway, by Nico Jungmann, are now on view at the Dowdeswell Galleries, 160 New Bond Street.

### Appointments.

Borough Small-Pox Hospital, Dewsbury.—Miss S. E. Turner has been appointed matron. She was trained at Marylebone Infirmary, London, and has since been nursing superintendent of the River Ambulance Service under the Metropolitan Asylums Board.

British Seamen's Hospital, Smyrna.—Miss Grace Williamson has been appointed superintendent, and Miss Frances S. Cooke assistant nursing sister. Miss Williamson was trained at the Derby Royal Infirmary, and has for the last three years held the post of assistant nursing sister of the British Seamen's Hospital, Smyrna. Miss Cooke was trained at Addenbrooke's Hospital, Cambridge, and has since held the post of sister at the Children's Hospital, Nottingham.

Glamorganshire County Council.—Mrs. S. A. Richards has been appointed lady inspector of midwives. She was trained at Epsom Infirmary, and for the past twelve months has been a charge nurse at the same institution. She holds the L.O.S. certificate.

Ilkley Hospital.—Miss L. E. Roffey has been appointed assistant matron. She was trained at Middlesex Hospital, London, and has since been sister at Reigate and Redhill Cottage Hospital. She has also done private nursing in Birmingham.

Jessop Hospital for Women, Sheffield.—Miss Martha Moore has been appointed staff nurse. She was trained at Warrington Infirmary, and has since been staff nurse in the same institution.

Linlithgow Poorhouse Combination. — Miss M. F. Campbell has been appointed charge nurse. She was trained at St. George's Infirmary, Fulham Road, London.

Milton Hospital, Portsmouth.—Miss Florence Mary Golding has been appointed sister. She was trained at St. Marylebone Infirmary, London, and has since been charge nurse at the Northern Hospital, Winchmore Hill, charge nurse at Craigleith Hospital, Edinburgh, nursing sister in the Concentration Camps, South Africa, and charge nurse at the South-Western Fever Hospital, Fulham, London.

Montrose Fever Hospital.—Miss E. J. Milne has been appointed matron. She was trained at Glasgow Royal Infirmary and Belvedere Fever Hospital.

Pontypridd Workhouse Infirmary.—Miss Ella Scott-Davies has been appointed charge nurse. She was trained at St. George's-in-the-East Infirmary, Wapping, London. She has since been staff nurse in the same institution.

Rochdale Infirmary.—Miss Ida Cruickshank has been appointed sister. She was trained at the Royal Infirmary, Newcastle-on-Tyne, and has since been head nurse at Tynemouth Infirmary, North Shields, sister at Stockton Hospital, and sister at Rotherham Hospital.

St. Dunstan's Infirmary, Fulham Road, London.—Miss Edith Amy Tucker has been appointed second assistant matron. She was trained at the Poplar and Stepney Sick Asylum, where she has since held the posts of staff nurse, ward sister, theatre sister, and night superintendent.

Southwark Infirmary, East Dulwich.—Miss Frances M. Tomkinson has been appointed sister. She was trained at the North Staffordshire Infirmary, Stoke-on-Trent, and has since been sister at the Royal Hospital, Richmond, Surrey.

Tynemouth Workhouse Infirmary.—Miss Caroline Berridge and Mrs. Mapstone have been appointed charge nurses. Miss Berridge, who is described as "untrained," has been charge nurse at Holborn, Wandsworth, and Clapham, Berkhamsted, Great Yarmouth, and Hensted Unions. Mrs. Mapstone was trained, for one year, at Grimsby and at the City of London Lying-in Hospital. She has since been on the staff of the Nurses' Home and Training School at Newcastle-on-Tyne and has done private nursing.

Wallingford Isolation Hospital.—Miss F. M. Birkin has been appointed matron. She was trained at Marylebone Infirmary, London.

Woodford Jubilee Hospital.—Miss L. M. Firth has been appointed matron. She was trained at Charing Cross Hospital, where she was afterwards sister. She has since been matron of Diss Cottage Hospital.

### Help the Nurses to Help Themselves.

East London Nursing Society.—The object of this society is to nurse the sick poor in East London in their own homes by means of trained resident nurses, each nurse living in the parish in which she works. In 1903 the staff of 27 nurses attended to 4,652 persons, to whom 115,209 visits were made. Annual subscriptions and donations to the general fund are asked for. Secretary, Mr. Arthur W. Lacey, 43 Rutland Street, New Road, Commercial Road East, E.

Queen Victoria's Jubilee Institute for Nurses.—The institute trains nurses in district nursing, and supplies nurses to affiliated associations for the sick poor in their own homes. Applications for information should be addressed to the General Superintendent. The offices are at 120 Victoria Street, S.W.

Colonial Nursing Association.—This valuable association was founded seven years ago to supply trained nurses to the Crown Colonies and small British communities in foreign countries. Since the foundation 238 nurses have been dispatched to various parts of the world, grants in aid being made where it is clearly shown to be impossible for the residents unassisted to bear entire cost of passage moneys, salaries, and maintenance. The Hon. Secretary, Miss C. A. Hutton, Imperial Institute, S.W., will be glad to receive contributions, especially as an effort is being made just now to extend its benefits to the poorer colonies.

Royal National Pension Fund for Nurses.—During 1904 close upon 1,200 policies were taken out in the Fund, a number 300 in excess of the highest previous total for twelve months. The year was marked by a liberal concession as regards surrender value of those policies which had been in existence seven full years; for it was decided that a nurse who withdrew her premium under a policy which had been in force that period and over, should receive the full accumulated interest of two and a half per cent. per annum without any deduction for expenses of administration. A considerable number of policy-holders entered upon their annuities, and the amount paid out under this head was £12,000 as compared with £10,000 in 1903. Sick pay to the extent of £1,650 was distributed amongst members who had become incapacitated through illness from following their employment. The income from all sources was £128,000 and the invested funds reached the grand total of £900,000. In July her Majesty, the President, presented certificates at Buckingham Palace to those nurses who had entered the Fund since the previous similar reception in 1901. Offices, 28 Finsbury Pavement, London, E.C.

### Echoes from the Outside World.

### Movements of Royalty.

On Saturday the King and Queen and the Prince and Princess of Wales returned to London in order to take leave of the King of Portugal, who, after a stay in England of nearly a month, left the metropolis in the afternoon for Paris, having previously lunched with their Majesties and other members of the Royal Family at the Portuguese Legation. The Queen of Portugal's visit was unfortunately shortened by the serious illness of her sister, the Duchess of Aosta. The latter has, however, so far recovered that the Queen was able to join her husband in Paris on Sunday. King Edward and the Prince of Wales accompanied King Carlos to Victoria Station on Saturday, and the Royal party received an enthusiastic ovation from a large crowd. The luggage of King Carlos comprised 190 pieces, mostly very large cases containing purchases, which His Majesty was taking back with him.

On Monday King Edward, in the capacity of sponsor, attended at the baptism of the infant son and heir of the Duke of Westminster at the Chapel Royal, St. James's Palace, the child receiving the name of Edward George Hugh. The King signed the register, and his gift to the infant was a two-handled silver cup and cover. The King and Queen left town on Monday afternoon for Culford Hall, Bury St. Edmunds, the seat of Lord Cadogan. On their arrival at Culford the men engaged on the estate were ranged with torches on either side of the drive from the chief entrance to the hall.

### Gift by the King of Portugal to the British Nation.

The British nation is indebted to the King of Portugal for an important collection of fishes caught off the coast of Portugal by his Majesty himself. The presentation was made to the trustees of the British Museum before the King's departure from England, and the series consists chiefly of fishes of large size, including several sharks and dog-fishes, one of the most noteworthy examples being an eel-shaped shark. The King is an enthusiastic ichthyologist of a very high order, and a number of illustrated scientific publications embodying the results of his researches and investigations made during his numerous cruises on board his yacht *Amelia* are included in his donation.

### The War in the Far East.

On Saturday the Japanese Legation in London received a telegram from the commander of the Naval Artillery Corps at Port Arthur in which he gave particulars of the incapacitation of the whole Russian Fleet. It was announced on Monday that a Japanese cruiser of over 1,300 tons, which was engaged in the blockade of the port, struck a Russian mechanical mine on November 30, and sank. From the front on the Sha-ho it is reported that the cold is becoming more intense. On Saturday night the thermometer fell to six degrees below zero. It is officially announced at Tokio that there have been further heavy casualties amongst the Japanese officers before Port Arthur. A statement is current in St. Petersburg that General Kuropatkin has decided not to take the offensive until February, when his three armies will be completely organised.

### The Tsar's Gift to British Officers.

The Tsar has presented a silver bowl and ladle to the ward-room officers of the British warship *Talbot*, for their services to the crews of the Russian warships engaged at the battle of Chemulpo, and also a cheque for £500 to the Prince of Wales, as President of the Royal Naval Fund, to which this money is destined, in recognition of the *Talbot's* services. The receipt of the bowl, and of the cheque has been cordially acknowledged, and his Imperial Majesty has been informed that the bowl will be "a lasting memento of an occasion on which the officers and crew of the *Talbot* were fortunately able to afford assistance to their fellow-sailors in distress."

### Queen Draga's Jewels and Costumes.

The sale of the jewels and costumes of the late Queen Draga of Servia attracted an enormous number of people, though there were only seven lots and the whole of the articles were disposed of in 14 minutes. For a brilliant tiara with two large brilliants in the centre, which the unfortunate Queen wore at her wedding, £1,220 was given. An emerald and brilliant bracelet, which formed part of the necklace sent as a wedding present to the Queen by the Tsar, fetched £480. The Persian Order of the Sun, presented to the Queen by the Shah of Persia on the occasion of his visit to Belgrade in 1900, realised £115. The Order is of the greatest rarity, being presented only to Sovereign ladies. The Turkish Order of Nischani-Schefkat was sold for £100. The Queen's bridal gown, of soft ivory satin, worked into tucks, and profusely trimmed with narrow flounces and insertions of Bruges lace, was knocked down at £30; and her State costume, a very elaborate robe of royal purple velvet, and including a tiara of gold, set with rubies, turquoise, pearls, and diamonds, at £270. The final lot, a gold pendant and pair of earrings, of Servian design, set with pearls and diamonds, went for £70.

### The New Bishops.

The King has approved the appointment of Dr. Charles Gore as first Bishop of Birmingham, Dr. Huyshe Yeatman-Biggs as Bishop of Worcester, and the Ven. J. W. Diggle as Bishop of Carlisle. Dr. Gore, who is 51, has been Bishop of Worcester since the resignation of the late Dr. Perowne in 1902, and, it is no secret, has, at his own desire, been transferred to the new see. He is unmarried. Dr. Yeatman-Biggs, who is 59, was trained for holy orders by the late Dean Vaughan, became vicar of St. Bartholomew's, Sydenham, in 1879, and in 1891 was appointed Suffragan Bishop of Rochester, taking the title of Bishop of Southwark. His wife is Lady Barbara, daughter of the fourth Earl of Dartmouth, and his work in assisting to promote and endow the new South London bishopric is well known. Archdeacon Diggle, who is 57, is the elder brother of Mr. J. R. Diggle, former chairman of the London School Board, and commenced his career in Manchester. He was for several years vicar of Mossley Hill, Liverpool, and since 1901 rector of St. Martin's, Birmingham. He married, as his second wife, Miss Moss, of Aigburth, Liverpool.

### Squalid Baby-Schools.

In the course of a lecture delivered on Friday evening Miss K. Phillips, London County Council superintendent of infant schools, deplored the present condition of the instruction given to children of from three to five years of age, and said that while some baby-schools are delightful, the majority are squalid, dismal places where the poor infants are kept in a gallery from nine to twelve and two to four o'clock, the chief commandment being "Thou shalt not move." Miss Phillips dwelt on the injury which was inflicted on the sight of such young children by obliging them to hold pencils and write between lines, and affirmed that, under existing arrangements, they are made the victims of hard-and-fast school rules until they lose the form of self-adjustment and originality and become dull machines. She declared that they would be better in the gutter, where at least they could tumble freely and develop physically, which is impossible in their present surroundings.

### Christmas Books.

(Continued from page 160.)

WELLS, GARDNER, DARTON AND CO.

All girls will like "The Family Grievance," by Raymond Jackbern (1s. 6d.). The story deals with school and home life, and is written in a bright, interesting manner. For a boy, especially one with a leaning towards colonial life, "Chris. Wharton" (1s) will be found thoroughly suitable. It belongs to the Chatterbox series. In "Wings," by Stella Austin, we recognise an old friend. It is now published, well bound in cloth, and nicely printed, for the small sum of 6d. The new edition of "Mrs. Leicester's School," by Charles and Mary Lamb, is wonderfully cheap at 1s., and contains one or two charming illustrations. For the tiny nursery folks, there should be a demand for "The Doll Book" (1s.), written and illustrated by May Gladwin, as it has the merit of originality. A most fascinating coloured picture of a little boy with a red parasol protecting a little girl against a flock of geese is a pleasant harbinger of bright reading in big print to be found in "Leading Strings" (1s. 6d ).

WARD LOCK AND CO.

There is never any risk in presenting a boy with a book by Gordon Stables, because he is certain to like it. "In Regions of Perpetual Snow" (5s.), the first scene is in the Highlands, and the story is continued in the trapping grounds of the far West, where there are battles with Indians and with wolves, and other adventures dear to the heart of a schoolboy. The hero at the end wins the V.C. in the South African war. "The Thrall of Leif the Lucky" (5s.) also deals with the scenery of northern lands, but instead of modern times Ottilie A. Liljencrantz has chosen the tenth century, and woven a most interesting and stirring story around the old Vikings. The illustrations are by Troy and Margaret West Kinney. By the same author and illustrated by the same artists is "The Ward of King Canute" (5s.). This is an historical romance of the best kind, and should be read with equal pleasure by boys and girls of 15 or 16 and by their mothers and fathers. There are real power and grit in Mr. Justus Miles Forman's romance "The Garden of Lies" (6s.), which has already reached a fourth edition. The interest is augmented by several excellent illustrations.

ADAM AND CHARLES BLACK.

Mr. S. R. Crockett's contribution to Christmas literature this year is a new departure. He calls it "Red Cap Tales" (6s.), and the stories are written for children who will not read Scott for themselves. There are tales from "Waverley," Guy Mannering," "Rob Roy," and "The Antiquary," and the young readers will probably end in loving the "Wizard of the North," as much as does the author himself. Quite different in style is "The Rat" (6s.), by G. M. A. Hewett, the first of a series of animal autobiographies. It is an amusing book with quaint sidelights upon the lives of human beings as seen from the little rodent's point of view. "The Dog" (6s.) has succeeded, by the help of G. E. Mitton, in giving to the public a delightful book.

HURST AND BLACKETT, LIMITED.

"The Marriage Yoke" (6s.), by Arabella Kenealy, appeals especially to nurses. The heroine is herself a nurse, who has to give up hospital work on account of her health, but goes away to the West of England to attend on a private patient with the hope of returning. She returns, but many pages are occupied with the remarkable things which happen to her in the interval.

MACMILLAN AND CO.

"The Ruby Ring" (4s. 6d.) is by Mrs. Molesworth, and the illustrations are by Miss Rosie Pitman. The story is that of a little girl who, not content with her own position, is enabled by means of a ring to become in turn a gipsy, a robin, and a fairy. The result of these experiences is a happier frame of mind on the part of little Sylvia.

C. ARTHUR PEARSON.

To children who reckon the Wallypug among their personal friends, and look forward to something fresh about him year by year, Mr. G. E. Farrow's last account of its doings entitled "The Wallypug in Fogland" (5s ) will be particularly welcome. Mr. Archibald Williams has described, in "The Romance of Modern Locomotion" (5s.), in non-technical language how railways are built, how life is protected in the signal box and by brakes, what expresses can do, and the part that the railway plays in war.

WALTER SCOTT PUBLISHING COMPANY.

Lovers of fairy lore have been particularly well catered for this year; "Swedish Fairy Tales" (6s.) written by F. Berg and translated by Tyra Engdahl and Jessie Rew is a beautiful book bound in white and crimson, and printed luxuriously on thick paper with many illustrations.

SWAN, SONNENSCHEIN AND CO.

"John Rigdon" (6s), by Charles P. Plant, is a strong novel, with much stirring action and an original plot. The American hero owing to a turn in the wheel of fortune suddenly finds himself transformed from a rich free man happily engaged to be married into a penniless slave, who is sold at once by the claimants of his father's property. The story of the fight which he makes against circumstances is well told, and is full of interest. Admirers of the late Clifford Harrison will welcome his "Hints to Reciters" (1s. 6d.), with a preface by Herbert Hardinge. "The Shakespeare Story" (1s. 6d.) is dedicated to the Education Committee of the London County Council.

WILLIAM HEINEMANN.

Mr. Hall Caine's latest romance, "The Prodigal Son" (6s.), is certain to be in demand for a Christmas present. Although but little happiness is meted out to the characters, who are exceedingly human in their failings, the pages of the book are enlivened by the devotion of Aunt Margret, the sweetness of the poor little wife Thora, and by the glowing descriptions of the Iceland scenery amongst which many of the scenes are passed.

T. FISHER UNWIN.

"They Twain" (6s.), by Mrs. Aubrey Richardson, is a novel which deals with married life. The husband's actions are misconstrued by his young wife; there are some months of estrangement, followed at the end by a happy reconciliation. The latest work by Lucas Cleeve "Children of Endurance" (6s.), contains the life and history of a "latter-day prophet," and would be of especial interest to those who espouse the cause of Judaism.

GEORGE A. MORTON, EDINBURGH.

MR. FREDERICK GRAVES, the author of "Ombra the Mystery" (6s.) treats his readers to some rather bloodcurdling scenes, but he also maintains their interest in the development of a quite original story, with many pretty scenes, and a good deal of pleasant dialogue. He certainly knows Edinburgh well, and the descriptive portions of his book are not the least admirable feature.

### Notes and Queries.

**FOR REGULATIONS SEE PAGE 12.**

*Meals for Epileptics.*

(79) Can you tell me of any book which would be a help in choosing meals for an epileptic patient whose digestion is rather poor at the best. I have "The Art of Feeding the Invalid," but epilepsy is not mentioned?—*R. S.*

There is no special diet, but a non-stimulating one is indicated. If any difficulty occur, the medical man should be consulted.

*L.O.S.*

(80) Will you kindly tell me if fully-trained nurses are required by the Central Midwives Board to train for three or six months for the L.O.S. certificate? Also if there is a nursing home in Cheltenham where nurses are received for preparation for the L.O.S. certificate?—*Equipped.*

The Central Midwives Board has nothing to do with the conditions under which the London Obstetrical Society grants certificates. The London Obstetrical Society requires proof of the candidate having attended 20 labours, and having been satisfactorily instructed in theoretic work, but it lays down no restrictions as to the time occupied in acquiring the necessary knowledge. There is no maternity nursing school in Cheltenham, but perhaps the Victoria District Nursing Association of Cheltenham Nurses can assist you.

I hold the certificate for the L.O.S., dated October 1898. I am now told that it is valueless and that I must pay 12s. 6d. for a new certificate. Will you kindly tell me if a certificate paid for in that way will qualify me to practise as a midwife?—*Sister L.O.S.*

The Secretary of the Association for Promoting the Training and Supply of Midwives, Dacre House, New Tothill Street, London, S.W., will give you every information.

*Home.*

(81) Will you kindly give me the name of a home for old ladies who have a little money, but not enough to pay for lodgings and to live upon when sickness comes? I believe there is one in London or the suburbs.—*M. J. C.*

You will find several mentioned in "Homes for Gentlewomen," sold by the Army and Navy Stores, price 1s.

*Massage.*

(82) Will you kindly tell me which is the best place to learn massage, what length of time it takes, what is the fee, and if there is an examination? I am a trained nurse.—*M. F.*

The School for Massage at the National Hospital for the Paralysed and Epileptic, Queen Square, Bloomsbury, W.C., is amongst the best. Write for particulars to the Matron.

*Hospital Training.*

(83) I wish to train as an army nurse when I am old enough. I am 19 and would like to know if it would be of any advantage to train in a children's hospital first as I am anxious to begin at once. Is St. Thomas's a good hospital at which to train for the military or naval nursing service?—*O. C.*

You had much better wait until you are 21 before commencing to train. St. Thomas's is a first-rate training school.

*Home.*

(84) Will you kindly tell me if there is a home in either Cornwall or Dorset where a patient in the first stage of phthisis could be received? I am making inquiries for a lady whose son has been ordered to either of the counties mentioned.—*Queen's Nurse.*

Procure "List of Sanatoria," published by the National Association for the Prevention of Consumption, from the Scientific Press, Southampton Street, Strand.

*Knitting.*

(85) I should like to get some knitting done for charitable purposes. Can you suggest a blind school or any place where such work is undertaken.—*Wellington.*

The Secretary, the British and Foreign Blind Association, 206 Great Portland Street, W., would doubtless be glad to recommend workers, or the matrons of other institutions for the blind.

**Handbooks for Nurses.**

| | Post Free. |
|---|---|
| How to Become a Nurse: "The Nursing Profession" ... | 2s. 4d. |
| "Nurses' Pronouncing Dictionary." Terms and Treatment... | 2s. 0d. |
| "A Handbook for Nurses." Dr J. K. Watson ... | 5s. 4d. |
| "Elementary Physiology for Nurses" ... | 2s. 0d. |
| "Swedish Massage and Movements" ... | 2s. 6d. |

Of all booksellers or of The Scientific Press, Limited, 28 & 29 Southampton Street, Strand, London, W.C.

### For Reading to the Sick.

### IMMORTALITY.

For ever and for ever
 The eternal mountains rise,
And lift their virgin snows on high
 To meet the silent skies.
Yet shall this soul which measures all,
While these stand steadfast, sink and fall?

For ever and for ever
 The swift suns roll through space;
From age to age they wax and wane,
 Each in its ordered place:
Yet shall this soul, whose inner eye
Foretells their cycles, fade and die?

For ever and for ever
 We have been, and we are,
Unchanging as the ocean wave,
 Unresting as the star:
Though suns stand still, and time be o'er,
We are, and shall be, evermore.

*Lewis Morris.*

The immortal soul is self-conscious. It is conscious too of universal life, and of its own place in it. . . . It is satisfied with the knowledge of the fact of mystery, as that without which eternity would be an idle void. It listens to the echoes of a distant past. It has never let go the hand of God. It sees and hears His guiding spirit in the trial sphere of human life. It knows His footsteps: it sees His light; it traces Him in the excellence and beauty of the universe, for all Nature is His parable.—*T. Gambier Parry.*

It is the steadfast self-realisation of a personal spirit which gives the true sanctity to this life, and reaches with irresistible confidence beyond the death wherein it hardly seems to die. In that which each one of us means, or should mean, when he says "I," there is already latent the prophecy of a personal immortality, and the assurance that though worms destroy this body, "yet in my flesh shall I see God; Whom I shall see for myself, and mine eyes shall behold, and not another."

It is when we recall ourselves from the scattered activity of our daily life; it is either when we have courage to go apart and stand alone and hear what the Lord God will say concerning us, or else when sickness or age has forced us into the solitude which we have always shunned: it is then that we know ourselves, and our need of a sufficient object in which the life of the soul may find its rest for ever. As one after another the interests of this world are removed; as we begin to see that our work is not an adequate or life-long scope for all we are, as the friends whom God has given us go before us into death, and one by one the channels of our love are closed; we may feel at last that deep, mysterious energy of the soul which must be set towards another Spiritual Being like itself.—*Dean Paget.*

Swift to its close ebbs out life's little day,
Earth's joys grow dim, its glories pass away,
Change and decay in all around I see,
O, Thou who changest not, abide with me.

*Henry F. Lyte.*

